# Evaluating the Role of Intraoperative Voiding Score in Prognostication Following Transurethral Resection of the Prostate

**DOI:** 10.7759/cureus.87207

**Published:** 2025-07-03

**Authors:** Muhammad Raheel, Sadaqat Ibrar, Shehryar Khan, Muhammad Moeed, Raza Muhammad, Muhammad Tayyib, Liaqat Ali, Muzzamil Sohail, Abdul Haseeb

**Affiliations:** 1 Department of Urology and Transplantation, Institute of Kidney Diseases, Hayatabad Medical Complex, Peshawar, PAK; 2 Department of Surgery, Hayatabad Medical Complex Peshawar, Peshawar, PAK

**Keywords:** bph, intraoperative void score, outcomes, successful trial without catheter, trans urethral resection of prostate (turp)

## Abstract

Introduction: Transurethral resection of the prostate (TURP) is an established treatment option for benign prostatic hyperplasia (BPH) with bladder outlet obstruction. Recently, the intra-operative void score (IVS) has emerged as a potential objective tool for assessing TURP efficacy by evaluating irrigating fluid flow. This study aimed to investigate IVS’s role in predicting outcomes of TURP.

Methodology: This prospective observational study was conducted at the Institute of Kidney Diseases, Peshawar, Pakistan, from January to March 2025. Forty male patients aged ≥50 years with BPH underwent TURP. IVS was assessed by emptying the bladder, instilling 300 mL of glycine irrigation solution, and applying a standardized 50 newton force to the suprapubic area and the Void score was calculated both pre-resection and post-resection. Patients were stratified into two groups based on combined IVS (high vs low combined IVS) and based on post-resection IVS (high vs low post-resection IVS) and compared for outcomes. Our primary outcomes included a successful trial without catheter (STWOC) and catheter-free follow-up (CFF). Univariate and multivariable analyses were conducted with a significance level set at p<0.05.

Results: This study examined 40 subjects who underwent TURP during the study period. The mean ± SD age was 63.8 ± 8.9 years, and the average prostate size was 70.5 ± 14.3 grams. Sixteen patients had a low combined IVS score, and 24 had a high combined IVS score. A total of 14 patients had a low post-resection IVS, and 26 had a high post-resection IVS. Among these groups, the study population demonstrated balanced baseline characteristics and similar comorbidity profiles (all p>0.05). Multivariable regression analysis demonstrated that high combined IVS had 3.2-fold greater odds of STWOC (aOR=3.2, 95% CI:1.6-6.4, p=0.001) and 3.9-fold greater odds of CFF at six weeks (adjusted odds ratio (aOR)=3.9, 95% CI:1.7-8.9, p=0.002). Similarly, high post-resection IVS was independently associated with improved outcomes, including 2.8-fold greater odds of STWOC (aOR=2.8, 95% CI:1.3-6.0, p=0.008), 3.1-fold greater odds of CFF (aOR=3.1, 95% CI:1.2-8.0, p=0.02).

Conclusion: IVS represents a promising tool for real-time assessment of TURP efficacy and prediction of postoperative outcomes. Higher combined and post-resection IVS were associated with significantly higher odds of STWOC and CFF, representing much better outcomes. This simple scoring system could potentially improve surgical decision-making and patient counseling regarding expected outcomes following TURP.

## Introduction

Benign prostatic hyperplasia (BPH) is a common nonmalignant but debilitating condition in men above 40 years of age, with the prevalence reaching more than 80% above the age of 80 years [1,2]. Among the several different treatment options, Transurethral resection of the prostate (TURP) remains the treatment of choice for BPH with severe bladder outlet obstruction (BOO) [3]. This procedure effectively opens the urinary passage, quickly relieving obstructive symptoms, like voiding difficulty [4]. The downside, however, includes failure of the procedure, persistence of symptoms, incontinence, and complications like the TURP syndrome, infections, strictures, fistulae, and sepsis [5,6]. Conventionally, post-void residual urine (PVR) and uroflowmetry are assessed post-operatively and can predict surgical success, but they lack real-time feedback during surgery [7].

The intra-operative void score (IVS) has recently gained consideration as a potential indicator of TURP effectiveness [8]. The intra-operative voiding score (IVS) measures the adequacy of prostate tissue resection by observing the flow of irrigating fluid during surgery. It is considered to be associated with post-operative outcomes, such as symptom improvement and a lower risk of complications [8]. However, the efficacy and reliability of IVS as a predictive tool remain underexplored, particularly in diverse clinical settings. IVS has the potential to fill this gap by providing surgeons with an intra-operative tool to evaluate resection adequacy, helping to minimize the risk of incomplete procedures and enhancing patient outcomes [4,5]. Additionally, recognizing the connection between IVS and post-operative symptoms, such as persistent storage issues or detrusor overactivity, could improve patient selection and optimize surgical planning [6,7].

This study aims to evaluate the role of IVS in predicting post-operative success following TURP in diverse patient settings like ours. This study aimed to determine whether IVS is a useful indicator of post-operative outcomes. It was hypothesized that higher IVS would be associated with a greater likelihood of successful trial without catheter (STWOC) and catheter-free follow-up (CFF) after TURP.

## Materials and methods

Study design, duration, and settings

This prospective observational study was conducted at the Institute of Kidney Disease (IKD), Peshawar, Pakistan, from February 1, 2025, to March 31, 2025. The study protocol received approval from the Institutional Research and Ethics Board of the Institute of Kidney Diseases (IKD), Peshawar, with approval number 521/Chairman/R&E/Committee/IKD, prior to commencement of the study. The study adhered to ethical guidelines, and written informed consent was obtained from all participants.

Inclusion and exclusion criteria

We included all male patients aged 50 years or older who were diagnosed with BPH, had an indwelling per-urethral catheter due to failed medical therapy, and were scheduled for monopolar TURP. Patients were excluded if they had a history of prostate cancer, bladder cancer, neurogenic bladder, previous prostate surgery, or if they refused to consent.

Sample size and sampling technique

The sample size was determined based on previous studies investigating the relationship between intraoperative void score (IVS) and TURP outcomes. With a 90% anticipated rate of STWOC in patients with high IVS (based on a pilot study), 80% power, a significance level of 0.05, and a confidence interval of 10%, the minimum required sample size was calculated to be 35 patients [9]. To enhance statistical power and account for potential exclusions, we enrolled 40 consecutive patients who met the inclusion criteria during the study period.

Data collection procedure

After approval from the Institutional Research and Ethical Board (IREB) and written informed consent from the patients or their legal surrogates, we enrolled the patients in our study based on our inclusion and exclusion criteria. Data was collected pertaining to baseline biographic and demographic characteristics (age, residence status), comorbid conditions (diabetes mellitus (DM), hypertension (HTN), ischemic heart disease (IHD), congestive heart failure (CHF), chronic kidney disease (CKD), chronic liver disease (CLD), chronic obstructive lung disease (COPD), body mass index (BMI), and obesity (BMI>30 kg/m^2^). We also collected data on pre-operative and post-operative prostate size on transabdominal ultrasound, post-void residual volume, maximum urinary flow rate (QMax) via uroflowmetry, voiding score (intra-operative pre-resection, post-resection, and combined IVS), outcome measures (STWOC, CFF), complications, and mortality.

All TURP procedures were performed by experienced urologists using standard monopolar resection techniques under spinal or general anesthesia based on anesthetic risk assessment and institutional protocols. Anesthesia type was documented and included in the baseline characteristic tables. Its association with postoperative outcomes, including STWOC, was analyzed. During surgery, the IVS was assessed before and after resection. A standardized suprapubic pressure of 50 Newtons was applied using a calibrated digital non-spring scale. Two independent assessors (the operating surgeon and first assistant) evaluated the fluid flow through the prostatic urethra using a predefined 6-point scale (0 = no flow, 1 = dribble, 2 = poor, 3 = moderate, 4 = good, 5 = excellent). The assessment was performed twice for consistency, with any discrepancies resolved through discussion to reach a consensus score. Following the evacuation of resected tissue, 300 mL of glycine irrigation solution was instilled into the bladder through the cystoscope, and again, the void score was estimated using the same method (post-resection IVS). Pre- and post-resection IVS ranged from 0 to 5. As introduced by earlier studies, we also calculated the combined IVS by adding the post-resection IVS and the change in IVS (pre-resection minus post-resection score), ranging from 0 to 10.

Postoperatively, the urethral catheter was typically removed on the first postoperative day. Patients were monitored for successful voiding trial outcomes and followed up at six weeks to assess long-term catheter-free status and symptom improvement. Any postoperative complications during this time were also recorded.

Patient stratification

Participants were mainly stratified into two groups based on their combined IVS (low combined IVS: 0-6 versus high combined IVS: 7-10) for comparison of baseline characteristics and outcomes. The cut-off values for low versus high combined IVS (0-6 vs. 7-10) were selected based on a previous study [8]. Sub-analyses were carried out using a second categorization based on the post-resection IVS (low post-resection IVS: 0-3 vs. high post-resection IVS: 4-5), and these two groups were also compared for outcomes.

Outcomes

Our primary outcome was a successful trial without catheter (STWOC), meaning no requirement for re-catheterization within 24 hours of catheter removal, and catheter-free follow-up (CFF), defined as no episodes of acute urinary retention necessitating catheterization during the six-week follow-up period. Post-operative improvement in lower urinary tract symptoms was assessed using a subjective BPH Symptom Scale ranging from 1 to 10, with higher scores indicating more severe symptoms. Patients were asked to rate their symptoms preoperatively and at the one-month follow-up. The median score and interquartile range (IQR) were reported for each group. Secondary outcomes included improvements in symptoms of BPH, reduction in post-void residual urine volume, postoperative complications, and in-hospital mortality. 

Statistical analyses

All statistical analyses were performed with SPSS software, version 25 (IBM Corp., Armonk, NY). The groups were compared for baseline characteristics, outcomes, and complications in univariate analyses. Continuous variables were presented as means ± standard deviation (SD) for parametric data and as medians and interquartile ranges (median, IQR) for non-parametric data. Categorical variables were reported as frequencies and percentages, and the groups were compared for the proportions of these categorical variables using univariate tests like Chi-square and Fisher’s exact test, while an independent sample t-test was conducted to compare the means of the qualitative variables. Multivariable logistic regression models were used to identify the independent association of combined IVS and post-operative IVS with STWOC and CFF. A p-value <0.05 was considered statistically significant for all analyses.

## Results

We analyzed the data of 40 patients undergoing TURP during the data collection period, of which 16 had a low combined IVS score and 24 had a high combined IVS score. Among the subjects, the mean ± SD age observed was 63.8 ± 8.9 years, with a mean ± SD BMI of 27.3 ± 3.8 kg/m^2^, and mostly an urban population (70%). Comparative analysis revealed no significant differences between Low and High combined IVS groups in age, BMI, the distribution of urban/rural residence, or comorbidity profiles, prostate size, Pre-operative void score, and the type of anesthesia received (all p>0.05) (Table 1). 

**Table 1:** Comparison of baseline characteristics of patients with Low and High combined intra-operative voiding scores Low IVS: Combined Intra-operative Void Score <6, High IVS: Combined Intra-operative Void Score ≥6. Body Mass Index (BMI), Chronic Kidney Disease (CKD), Chronic Liver Disease (CLD), Diabetes Mellitus (DM), Hypertension (HTN), Interquartile Range (IQR), Ischemic Heart Disease (IHD), Standard Deviation (SD), n (%): frequency (percentage), Obesity (BMI ≥30), prostate size and Pre-op void score. Continuous variables are reported as mean ± SD unless otherwise specified. Categorical variables are reported as frequency (percentage). † Mann-Whitney U test used for non-normally distributed ordinal variables. ‡ Fisher’s Exact Test used for categorical variables with expected cell counts <5 (e.g., CLD, mortality). The p-values were calculated using two-tailed tests with α=0.05. Significant results (p < 0.05) are highlighted in bold.

Variable	Total (n=40)	Low Combined IVS (n=16)	High Combined IVS (n=24)	p-value	Test Used
Age (years), mean ± SD	63.8 ± 8.9	65.4 ± 9.2	62.7 ± 8.6	0.35	Independent t-test
Residence, n (%)					
Urban	28 (70.0%)	10 (62.5%)	18 (75.0%)	0.49	χ²=0.48 (Chi-square)
Rural	12 (30.0%)	6 (37.5%)	6 (25.0%)		
BMI (kg/m²), mean ± SD	27.3 ± 3.8	28.1 ± 4.2	26.8 ± 3.5	0.29	Independent t-test
Comorbidities, n (%)					
Diabetes Mellitus	14 (35.0%)	7 (43.8%)	7 (29.2%)	0.35	χ²=0.88 (Chi-square)
Hypertension	22 (55.0%)	10 (62.5%)	12 (50.0%)	0.43	χ²=0.62 (Chi-square)
Ischemic Heart Disease	8 (20.0%)	4 (25.0%)	4 (16.7%)	0.69	Fisher’s exact test
Chronic Kidney Disease	5 (12.5%)	3 (18.8%)	2 (8.3%)	0.37	Fisher’s exact test
Chronic Liver Disease	2 (5.0%)	1 (6.3%)	1 (4.2%)	1.00	Fisher’s exact test
Obesity (BMI ≥30)	12 (30.0%)	6 (37.5%)	6 (25.0%)	0.49	χ²=0.48 (Chi-square)
Prostate Size (grams), mean ± SD	70.5 ± 14.3	72.3 ± 15.0	69.5 ± 14.0	0.56	Independent t-test
Pre-op void score, mean ± SD	1.7 ± 0.96	1.6 ± 0.84	1.8 ± 1.03	0.55	
Anesthesia Type, n (%)					
Spinal	24 (60.0%)	10 (62.5%)	14 (58.3%)	0.76	χ²=0.10 (Chi-square)
General	16 (40.0%)	6 (37.5%)	10 (41.7%)		

A total of 14 patients had a low post-resection IVS, and 26 had a high post-resection IVS. Among these groups, the study population demonstrated balanced baseline characteristics with no statistically significant differences in mean age, BMI, prostate size, Pre-op void score, and the type of anesthesia received (p>0.05). These groups also had similar comorbidity profiles with around equal proportions of diabetes mellitus, hypertension, obesity (p=0.72), and other less common conditions like CKD, CLD, and IHD. (p>0.05) (Table 2). 

**Table 2:** Comparison of baseline characteristics of patients with Low and High post-resection intra-operative voiding scores Low pre-resection IVS: Void Score <4, High pre-resection IVS: Void Score ≥4. Body Mass Index (BMI), Chronic Kidney Disease (CKD), Chronic Liver Disease (CLD), Diabetes Mellitus (DM), Hypertension (HTN), Interquartile Range (IQR), Ischemic Heart Disease (IHD), n (%): frequency (percentage), Obesity (BMI ≥30), Standard Deviation (SD). Continuous variables are reported as mean ± SD unless otherwise specified. Categorical variables are reported as frequency (percentage). † Mann-Whitney U test used for non-normally distributed ordinal variables. ‡ Fisher’s Exact Test used for categorical variables with expected cell counts <5 (e.g., CLD, mortality). The p-values were calculated using two-tailed tests with α=0.05. Significant results (p < 0.05) are highlighted in bold.

Variable	Total (n=40)	Low Post-resection IVS (n=14)	High Post-resection IVS (n=26)	p-value	Test Used
Age (years), mean ± SD	63.8 ± 8.9	65.1 ± 9.3	63.1 ± 8.7	0.52	Independent t-test
Residence, n (%)					
Urban	28 (70.0%)	9 (64.3%)	19 (73.1%)	0.72	Fisher’s exact test
Rural	12 (30.0%)	5 (35.7%)	7 (26.9%)		
BMI (kg/m²), mean ± SD	27.3 ± 3.8	27.9 ± 4.1	27.0 ± 3.6	0.48	Independent t-test
Comorbidities, n (%)					
Diabetes Mellitus	14 (35.0%)	6 (42.9%)	8 (30.8%)	0.50	χ²=0.46 (Chi-square)
Hypertension	22 (55.0%)	9 (64.3%)	13 (50.0%)	0.52	χ²=0.41 (Chi-square)
Ischemic Heart Disease	8 (20.0%)	3 (21.4%)	5 (19.2%)	1.00	Fisher’s exact test
Chronic Kidney Disease	5 (12.5%)	2 (14.3%)	3 (11.5%)	1.00	Fisher’s exact test
Chronic Liver Disease	2 (5.0%)	1 (7.1%)	1 (3.8%)	1.00	Fisher’s exact test
Obesity (BMI ≥30)	12 (30.0%)	5 (35.7%)	7 (26.9%)	0.72	χ²=0.13 (Chi-square)
Prostate Size (grams), mean ± SD	70.5 ± 14.3	72.3 ± 15.0	69.5 ± 14.0	0.56	Independent t-test
Pre-op void score, mean ± SD	1.7 ± 0.96	1.6 ± 0.84	1.8 ± 1.03	0.55	Independent t-test
Anesthesia Type, n (%)					
Spinal	24 (60.0%)	9 (64.3%)	15 (57.7%)	0.76	χ²=0.10 (Chi-square)
General	16 (40.0%)	5 (35.7%)	11 (42.3%)		

Univariate comparison of outcomes

Patients with high combined IVS demonstrated significantly better postoperative outcomes compared to those with low combined IVS, with markedly higher rates of STWOC (95.8% vs. 37.5%, p<0.001) and were more likely to remain catheter-free at six weeks (100% vs. 62.5%, p=0.001). Patients with higher IVS scores demonstrated significantly better postoperative symptom relief. The median [IQR] BPH symptom score at follow-up was 3 (2-4) in the high IVS group compared to 6 (5-7) in the low IVS group (p < 0.001), indicating greater symptom improvement among those with higher voiding scores. Symptom resolution at one month was also superior in the High combined IVS group (95.8% vs. 56.3%, p=0.003), and complications were substantially lower in this group compared to lower combined IVS (4.2% vs. 43.8%, p=0.005), with fewer urinary tract infections (4.2% vs. 25.0%, p=0.07) and no bleeding events (0% vs. 12.5%, p=0.15) (Table 3). 

**Table 3:** Comparison of outcomes of patients with Low combined and High combined intra-operative voiding scores Benign Prostatic Hyperplasia (BPH), Catheter Free Follow-up (CFF in six Weeks), Interquartile Range (IQR), Successful Trial Without Catheter (STWOC), Urinary Tract Infection (UTI). All Data are reported as n(%) unless otherwise specified. The p-values were calculated using two-tailed tests with α=0.05. Significant results (p < 0.05) are highlighted in bold.

Outcome Measure	Total (n=40)	Low Combined IVS (n=16)	High Combined IVS (n=24)	p-value	Test Used
Surgical Outcomes					
STWOC	29 (72.5%)	6 (37.5%)	23 (95.8%)	<0.001	Fisher's Exact
CFF	34 (85.0%)	10 (62.5%)	24 (100%)	0.001	Fisher's Exact
Clinical Outcomes					
BPH Symptom Scale (1–10), median [IQR]	4 [3–6]	6 [5–7]	3 [2–4]	<0.001	Mann–Whitney U
BPH Symptom Resolution at 1 Month	32 (80.0%)	9 (56.3%)	23 (95.8%)	0.003	Fisher's Exact
Total Complications	8 (20.0%)	7 (43.8%)	1 (4.2%)	0.005	Fisher's Exact
UTI	5 (12.5%)	4 (25.0%)	1 (4.2%)	0.07	Fisher's Exact
Bleeding Requiring Intervention	2 (5.0%)	2 (12.5%)	0 (0%)	0.15	Fisher's Exact
Stricture Formation	1 (2.5%)	1 (6.3%)	0 (0%)	0.40	Fisher's Exact
Readmission within 30 Days	3 (7.5%)	3 (18.8%)	0 (0%)	0.04	Fisher's Exact
Hospital Stay (days), mean ± SD	2.1 ± 0.8	2.4 ± 0.9	1.9 ± 0.6	0.06	Independent t-test

Patients with High post-resection voiding scores demonstrated significantly better clinical outcomes compared to those with Low post-resection scores. The High post-resection IVS group had markedly increased rates of STWOC (88.5% vs. 42.9%, p=0.003) and CFF at six weeks (92.3% vs. 71.4%, p=0.04). The median [IQR] BPH symptom score at follow-up was 4 (3-5) in the high IVS group compared to 6 (6-7) in the low IVS group (p < 0.001), indicating greater symptom improvement among those with higher voiding scores. Along with superior BPH symptom resolution at one month (88.5% vs. 64.3%, p=0.04). Total complications were also significantly lower in the High post-resection IVS group (11.5% vs. 35.7%, p=0.04), with a trend toward fewer urinary tract infections (7.7% vs. 21.4%, p=0.31). Additionally, the High IVS group experienced shorter mean hospital stays (1.9±0.6 vs. 2.5±1.0 days, p=0.02), though readmission rates were comparable (3.8% vs. 14.3%, p=0.27) (Table 4). 

**Table 4:** Comparison of outcomes of patients with Low and High post-resection intra-operative voiding scores Benign Prostatic Hyperplasia (BPH), Catheter Free Follow-up (CFF in six Weeks), Interquartile Range (IQR), Successful Trial Without Catheter (STWOC), Urinary Tract Infection (UTI). All Data are reported as n (%) unless otherwise specified. p<0.05 considered significant (two-tailed t-tests). Significant results (p < 0.05) are highlighted in bold.

Outcome Measure	Total (n=40)	Low Post-resection IVS (n=14)	High Post-resection IVS (n=26)	p-value	Test Used
Surgical Outcomes					
STWOC	29 (72.5%)	6 (42.9%)	23 (88.5%)	0.003	Fisher's Exact
CFF	34 (85.0%)	10 (71.4%)	24 (92.3%)	0.04	Fisher's Exact
Clinical Outcomes					
BPH Symptom Scale (1–10), median [IQR]	4 [3–6]	6 [6-7]	4 [3-5]	<0.001	Mann–Whitney U
BPH Symptom Resolution at 1 Month	32 (80.0%)	9 (64.3%)	23 (88.5%)	0.04	Fisher's Exact
Total Complications	8 (20.0%)	5 (35.7%)	3 (11.5%)	0.04	Fisher's Exact
UTI	5 (12.5%)	3 (21.4%)	2 (7.7%)	0.31	Fisher's Exact
Bleeding Requiring Intervention	2 (5.0%)	1 (7.1%)	1 (3.8%)	1.00	Fisher's Exact
Stricture Formation	1 (2.5%)	1 (7.1%)	0 (0%)	0.33	Fisher's Exact
Readmission within 30 Days	3 (7.5%)	2 (14.3%)	1 (3.8%)	0.27	Fisher's Exact
Hospital Stay (days), mean ± SD	2.1 ± 0.8	2.5 ± 1.0	1.9 ± 0.6	0.02	Independent t-test

Multivariable analyses

Multivariable regression analyses demonstrated that both high combined IVS had 3.2-fold greater odds of STWOC (aOR=3.2, 95% CI:1.6-6.4, p=0.001), 3.9-fold greater odds of CFF at six weeks (aOR=3.9, 95% CI:1.7-8.9, p=0.002), and 2.6-fold greater odds of BPH symptom resolution (aOR=2.6, 95% CI:1.3-5.4, p=0.009), while showing 60% lower odds of complications (aOR=0.4, 95% CI:0.2-0.9, p=0.03). Similarly, high post-resection IVS was independently associated with improved outcomes, including 2.8-fold greater odds of STWOC (aOR=2.8, 95% CI:1.3-6.0, p=0.008), 3.1-fold greater odds of CFF (aOR=3.1, 95% CI:1.2-8.0, p=0.02), and a significant reduction in hospital stay duration (β=-0.5 days, 95% CI:-1.0-0.0, p=0.049) (Table 5).

**Table 5:** Multivariable regression analyses for the independent association of high combined Intra-operative voiding score and high post-resection intra-operative voiding score with post-operative TURP outcomes aOR: Adjusted Odd’s ratio, CI: Confidence interval, TURP: Transurethral resection of the prostate. Regression analyses were performed while adjusting for age, gender, residence status, education level, time of surgery, and comorbid conditions. Significant results (p<0.05) are highlighted in bold. Beta coefficients (β) are shown for continuous outcomes such as hospital stay duration. All other reported values are adjusted odds ratios (aORs).

Variable	aOR/β	95% CI	p-value
For combined IVS			
Successful STWOC	3.2	1.6 – 6.4	0.001
Catheter-Free at 6 Weeks	3.9	1.7 – 8.9	0.002
BPH Symptom Resolution	2.6	1.3 – 5.4	0.009
Total Complications	0.4	0.2 – 0.9	0.03
Hospital Stay (days)	-0.4	-0.9 – 0.1	0.11
Readmission	0.3	0.1 – 0.9	0.04
For post-operative IVS			
Successful STWOC	2.8	1.3 – 6.0	0.008
Catheter-Free at 6 Weeks	3.1	1.2 – 8.0	0.02
BPH Symptom Resolution	2.3	1.1 – 4.9	0.03
Total Complications	0.5	0.2 – 1.2	0.11
Hospital Stay (days)	-0.5	-1.0 – 0.0	0.049
Readmission	0.4	0.1 – 1.8	0.23

## Discussion

This study demonstrated that the high combined IVS had over threefold higher odds of STWOC and CFF. Similarly, high post-resection IVS was independently associated with improved outcomes, including STWOC and CFF, and a significant reduction in the duration of hospital stay. These results confirm that both combined and post-resection IVS serve as robust independent predictors of surgical success and postoperative recovery following TURP, even after accounting for demographic and clinical variables. It also indicates that a higher post-resection voiding score strongly predicts better surgical success, faster recovery, and reduced complication rates following TURP, reinforcing its value as a prognostic indicator for postoperative outcomes. In addition, symptom improvement measured using a simplified BPH symptom scale (scored 1-10) showed significantly lower post-resection scores in high IVS groups, reflecting greater relief of urinary obstruction and improved patient-reported outcomes.

The baseline characteristics of our study population, including age, comorbidities, and prostate size, were comparable between groups stratified by IVS, ensuring minimal confounding bias. The mean age and comorbidity profiles align with prior studies examining TURP outcomes and reflect a typical cohort of elderly men with BPH [1]. Notably, the balanced distribution of diabetes, hypertension, and other comorbidities across IVS groups suggests that these factors did not significantly influence the observed outcomes. Likewise, there was no statistically significant difference in the distribution of anesthesia type (spinal vs. general) between IVS groups, indicating that anesthesia choice did not meaningfully affect postoperative outcomes such as STWOC or recovery. Previous research has similarly emphasized the importance of objective intraoperative metrics in TURP, though our study uniquely focuses on IVS as a dynamic, real-time assessment tool.

Wardill test is a conventional method of assessing the post-voidal flow where the urologist applies suprapubic pressure immediately after the resection and looks for the outflow of irrigation fluid [10]. A novel qualitative test called the Chambers test (after the urologist Roger Chambers from Auckland, United Kingdom) was recently introduced and tested to calculate the intra-operative void score [8]. In their study, Robinson et al. reported a high positive predictive value (100%) and high sensitivity (82%) for identifying STWOC with IVS [8]. If used accurately, this can be a very economical, real-time indicator and a highly reliable marker of outcomes of TURP. Such low-cost tests are especially advantageous in resource-limited settings like ours. We looked at this metric from a different angle, measuring the odds of successful outcomes with high IVS, and the results were equally fascinating. Robinson evaluated four types of void scores (pre-resection IVS, post-resection IVS, change in IVS, and combined IVS) and they saw the superiority of post-resection IVS and combined IVS for prognostication of TURP [8]. Our study findings reinforced these reports as both the high combined IVS and high post-resection IVS were associated with higher odds of improved outcomes after TURP.

Our findings offer advantages over traditional methods, such as the Wardill test described by Lynch and Stewart, which assesses voiding ability before catheter removal [10]. The IVS provides earlier predictions of outcomes during the procedure itself, potentially allowing for surgical alterations if needed rather than waiting until the postoperative period. Despite its real-time assessment, IVS is an intra-operative test and does not account for the post-operative changes in prostate and surrounding tissue, including any inflammation. IVS is also highly dependent on the assessors’ hands and experience. Therefore, we cannot recommend IVS as a replacement for traditional urodynamic studies but rather as a useful adjunct to them.

The strong link between a high combined IVS and improved outcomes likely reflects the combined influence of preoperative and intra-operative factors on surgical success. A higher combined score may indicate more effective resection of obstructive prostate tissue, resulting in improved urinary flow and less postoperative retention. This is consistent with the rationale that sufficient resection relieves bladder outlet obstruction and enhances voiding efficiency. The nearly fourfold higher odds of CFF in the high combined IVS group further suggest that this metric reflects both technical success and functional recovery. Once validated by large-scale studies, these numbers can even be used to educate and counsel our patients on the success of their surgery. It can also be used to construct a scoring system to indicate the requirement for any further intervention during or after surgery. This is beyond the scope of this study and should be tested in further prospective work.

Pre-operative urodynamic study is a tested method and reliable indicator of TURP outcomes and to predict its success [11]. While urodynamics remain the gold standard, our findings suggest that IVS could serve as a useful intraoperative tool to confirm obstruction relief, especially in settings with limited access to urodynamic studies or inconclusive preoperative results [12,13]. Yet, a theoretical comparison may not be as accurate, and it is imperative to test IVS and Urodynamic studies side by side in prospective randomized trials and to compare the utility of the novel IVS with the gold standard.

Post-resection IVS emerged as a critical determinant of outcomes, likely because it directly reflects the immediate anatomical and functional changes achieved during surgery. A high post-resection score signifies sufficient relief of obstruction, which correlates with faster recovery and lower morbidity. Complications like persistent obstructive symptoms and UTIs were notably lower in patients with high IVS. The inverse relationship between IVS and complications suggests that adequate resection minimizes residual obstructive tissue, reducing stasis and trauma, key contributors to postoperative morbidity. These findings align with prior studies that link incomplete resection to higher complication rates [14,15]. As they say, one person’s benefits are another person's side effects. While the significant improvement in obstructive symptoms is an obvious benefit, it may become a misery for individuals with weak pelvic floor muscles and old age [16]. Theoretically, such patients are at risk of urinary incontinence and are only prevented by obstructive prostatic tissue. With a successful TURP, they will be exposed to this new complication. IVS may be able to predict optimal (enough but not too extensive) resection of the prostatic tissue and may dictate to the surgeon when to stop further resection. This was not studied in our small sample study, and we recommend further trials to explore the potential of IVS to guide surgical precision and improve patient outcomes.

Limitations

There are a few limitations to our study, such as we had 40 patients in our sample, which is a small number. The follow-up period might not have been long enough to see all the outcomes. Also, we didn't compare IVS with comprehensive urodynamic studies, which are considered the best way to assess bladder function. Further studies with bigger groups of people and longer follow-up times are needed to confirm these results. The stratified groups in our analysis were not evenly sized (e.g., 16 vs. 24 and 14 vs. 26), as group assignment was based on observed IVS values rather than through randomization. Because patients were stratified based on their observed IVS scores rather than assigned randomly, there was an imbalance in group sizes, and there may be inherent differences in baseline characteristics. Additionally, our study did not assess patients' quality of life, which is an important dimension of post-TURP recovery. Future research should include validated QoL measures to offer a more complete understanding of patient outcomes.

## Conclusions

The intra-operative void score represents a promising tool for real-time assessment of TURP efficacy and prediction of postoperative outcomes. Higher postoperative IVS, greater change in IVS, and higher combined IVS all correlate significantly with both successful trials without catheter and maintained catheter-free status at follow-up. This simple scoring system could potentially improve surgical decision-making and patient counseling regarding expected outcomes following TURP.
